# *N*′-(2,3-Dimethoxy­benzyl­idene)-2-hydr­oxy-3-methyl­benzohydrazide

**DOI:** 10.1107/S1600536810012122

**Published:** 2010-04-02

**Authors:** You-Yue Han, Qiu-Rong Zhao

**Affiliations:** a Department of Chemistry and Life Science, Chuzhou University, Chuzhou, Anhui 239000, People’s Republic of China

## Abstract

In the title compound, C_17_H_18_N_2_O_4_, the dihedral angle between the two benzene rings is 6.0 (2)° and the mol­ecule adopts an *E* configuration with respect to the C=N bond. There is an intra­molecular O—H⋯O hydrogen bond in the mol­ecule, which generates an *S*(6) ring. In the crystal, mol­ecules are linked through inter­molecular N—H⋯O hydrogen bonds, forming *C*(4) chains running along the *c* axis.

## Related literature

For a related structure and background information, see: Han & Zhao (2010 ▶). For reference structural data, see: Allen *et al.* (1987 ▶).
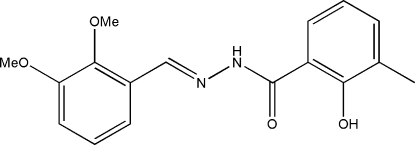


## Experimental

### 

#### Crystal data

C_17_H_18_N_2_O_4_
*M* *_r_* = 314.33
Orthorhombic, 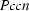

*a* = 14.923 (3) Å
*b* = 24.329 (5) Å
*c* = 8.7422 (17) Å
*V* = 3174.0 (11) Å^3^
*Z* = 8
Mo *K*α radiation
μ = 0.10 mm^−1^
*T* = 298 K
0.17 × 0.15 × 0.15 mm

#### Data collection

Bruker SMART CCD diffractometer
Absorption correction: multi-scan (*SADABS*; Bruker, 2001 ▶) *T*_min_ = 0.984, *T*_max_ = 0.986
17128 measured reflections
3461 independent reflections
1998 reflections with *I* > 2σ(*I*)
*R*_int_ = 0.223

#### Refinement

*R*[*F* ^2^ > 2σ(*F* ^2^)] = 0.067
*wR*(*F* ^2^) = 0.175
*S* = 0.92
3461 reflections
211 parameters
H-atom parameters constrained
Δρ_max_ = 0.23 e Å^−3^
Δρ_min_ = −0.30 e Å^−3^

### 

Data collection: *SMART* (Bruker, 2007 ▶); cell refinement: *SAINT* (Bruker, 2007 ▶); data reduction: *SAINT*; program(s) used to solve structure: *SHELXTL* (Sheldrick, 2008 ▶); program(s) used to refine structure: *SHELXTL*; molecular graphics: *SHELXTL*; software used to prepare material for publication: *SHELXTL*.

## Supplementary Material

Crystal structure: contains datablocks global, I. DOI: 10.1107/S1600536810012122/hb5386sup1.cif

Structure factors: contains datablocks I. DOI: 10.1107/S1600536810012122/hb5386Isup2.hkl

Additional supplementary materials:  crystallographic information; 3D view; checkCIF report

## Figures and Tables

**Table 1:** Hydrogen-bond geometry (Å, °)

*D*—H⋯*A*	*D*—H	H⋯*A*	*D*⋯*A*	*D*—H⋯*A*
O4—H4⋯O3	0.82	1.91	2.630 (2)	146
N2—H2*A*⋯O3^i^	0.90	2.17	3.030 (2)	158

Symmetry code: (i) 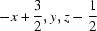.

**Fig. 1.**
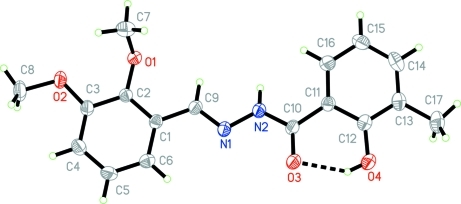
The molecular structure of (I) with 30% probability displacement ellipsoids for non-H atoms. Intramolecular O–H···O hydrogen bond is shown as a dashed line.

**Fig. 2.**
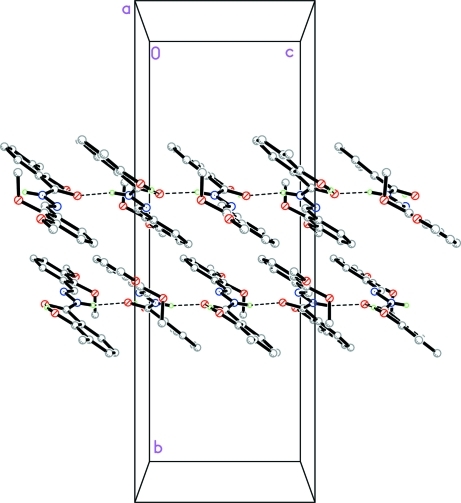
The molecular packing of the title compound, viewed along the a axis. Hydrogen bonds are shown as dashed lines.

## supplementary crystallographic information

### Comment

As part of our ongoing studies of hydrazones (Han & Zhao, 2010), we now report the structure of the title compound, (I).

In the molecule of the title compound, Fig. 1, the dihedral angle between the two benzene rings is 6.0 (2)°. The molecule adopts an *E* configuration with respect to the C═N bond. There is an intramolecular O–H···O hydrogen bond (Table 1) in the molecule. All the bond lengths are within normal ranges (Allen *et al.*, 1987).

In the crystal structure, molecules are linked through intermolecular N–H···O hydrogen bonds (Table 1) to form chains running along the *c* axis (Fig. 2).

### Experimental

A mixture of 2,3-dimethoxybenzaldehyde (0.166 g, 1 mmol) and 2-hydroxy-3-methylbenzohydrazide (0.166 g, 1 mmol) in 50 ml me thanol was stirred at room temperature for 1 h. The mixture was filtered to remove impurities, and then left at room temperature. After a few days, colourless blocks of (I) were formed.

### Refinement

H atoms were positioned geometrically and refined using the riding-model approximation, with C–H = 0.93 or 0.96 Å, O–H = 0.82 Å, N–H = 0.90 Å, and U_iso_(H) = 1.2U_eq_(C,*N*) or U_iso_(H) = 1.5U_eq_(methyl C and O).

### Crystal data

**Table:**

C_17_H_18_N_2_O_4_	*F*(000) = 1328
*M**_r_* = 314.33	*D*_x_ = 1.316 Mg m^−^^3^
Orthorhombic, *P**c**c**n*	Mo *K*α radiation, λ = 0.71073 Å
Hall symbol: -P 2ab 2ac	Cell parameters from 2994 reflections
*a* = 14.923 (3) Å	θ = 2.7–24.9°
*b* = 24.329 (5) Å	µ = 0.10 mm^−^^1^
*c* = 8.7422 (17) Å	*T* = 298 K
*V* = 3174.0 (11) Å^3^	Block, colorless
*Z* = 8	0.17 × 0.15 × 0.15 mm

### Data collection

**Table:**

Bruker SMART CCD diffractometer	3461 independent reflections
Radiation source: fine-focus sealed tube	1998 reflections with *I* > 2σ(*I*)
graphite	*R*_int_ = 0.223
ω scans	θ_max_ = 27.0°, θ_min_ = 1.6°
Absorption correction: multi-scan (*SADABS*; Bruker, 2001)	*h* = −18→18
*T*_min_ = 0.984, *T*_max_ = 0.986	*k* = −30→31
17128 measured reflections	*l* = −8→11

### Refinement

**Table:**

Refinement on *F*^2^	Primary atom site location: structure-invariant direct methods
Least-squares matrix: full	Secondary atom site location: difference Fourier map
*R*[*F*^2^ > 2σ(*F*^2^)] = 0.067	Hydrogen site location: inferred from neighbouring sites
*wR*(*F*^2^) = 0.175	H-atom parameters constrained
*S* = 0.92	*w* = 1/[σ^2^(*F*_o_^2^) + (0.0754*P*)^2^] where *P* = (*F*_o_^2^ + 2*F*_c_^2^)/3
3461 reflections	(Δ/σ)_max_ = 0.001
211 parameters	Δρ_max_ = 0.23 e Å^−^^3^
0 restraints	Δρ_min_ = −0.30 e Å^−^^3^

### Special details

**Table:**

Geometry. All esds (except the esd in the dihedral angle between two l.s. planes) are estimated using the full covariance matrix. The cell esds are taken into account individually in the estimation of esds in distances, angles and torsion angles; correlations between esds in cell parameters are only used when they are defined by crystal symmetry. An approximate (isotropic) treatment of cell esds is used for estimating esds involving l.s. planes.
Refinement. Refinement of *F*^2^ against ALL reflections. The weighted *R*-factor wR and goodness of fit *S* are based on *F*^2^, conventional *R*-factors *R* are based on *F*, with *F* set to zero for negative *F*^2^. The threshold expression of *F*^2^ > σ(*F*^2^) is used only for calculating *R*-factors(gt) etc. and is not relevant to the choice of reflections for refinement. *R*-factors based on *F*^2^ are statistically about twice as large as those based on *F*, and *R*- factors based on ALL data will be even larger.

### Fractional atomic coordinates and isotropic or equivalent isotropic displacement parameters (Å^2^)

**Table:**

	*x*	*y*	*z*	*U*_iso_*/*U*_eq_	
O1	0.42606 (9)	0.10101 (6)	0.37153 (16)	0.0482 (4)	
O2	0.28300 (9)	0.06224 (8)	0.52262 (16)	0.0602 (5)	
O3	0.84543 (10)	0.11847 (7)	0.62612 (16)	0.0586 (5)	
O4	1.01095 (9)	0.13449 (7)	0.53290 (16)	0.0601 (5)	
H4	0.9716	0.1232	0.5903	0.072*	
N1	0.67827 (11)	0.09279 (7)	0.53655 (18)	0.0464 (5)	
N2	0.74005 (11)	0.11742 (7)	0.44045 (19)	0.0474 (5)	
H2A	0.7274	0.1235	0.3411	0.057*	
C1	0.52468 (13)	0.06866 (8)	0.5673 (2)	0.0402 (5)	
C2	0.43791 (13)	0.07538 (9)	0.5098 (2)	0.0396 (5)	
C3	0.36482 (13)	0.05310 (9)	0.5889 (2)	0.0443 (5)	
C4	0.37887 (16)	0.02333 (10)	0.7208 (2)	0.0521 (6)	
H4A	0.3306	0.0079	0.7727	0.063*	
C5	0.46507 (16)	0.01651 (10)	0.7758 (2)	0.0536 (6)	
H5	0.4743	−0.0036	0.8649	0.064*	
C6	0.53705 (15)	0.03885 (9)	0.7014 (2)	0.0473 (5)	
H6	0.5945	0.0341	0.7406	0.057*	
C7	0.38589 (19)	0.15360 (11)	0.3766 (3)	0.0724 (8)	
H7A	0.3336	0.1523	0.4405	0.109*	
H7B	0.3690	0.1646	0.2751	0.109*	
H7C	0.4278	0.1797	0.4178	0.109*	
C8	0.20603 (16)	0.04196 (12)	0.6008 (3)	0.0701 (8)	
H8A	0.2071	0.0025	0.6009	0.105*	
H8B	0.1529	0.0546	0.5500	0.105*	
H8C	0.2063	0.0551	0.7043	0.105*	
C9	0.59903 (13)	0.09237 (9)	0.4826 (2)	0.0445 (5)	
H9	0.5885	0.1076	0.3867	0.053*	
C10	0.82117 (13)	0.13106 (9)	0.4951 (2)	0.0428 (5)	
C11	0.88015 (14)	0.16270 (8)	0.3910 (2)	0.0421 (5)	
C12	0.97309 (14)	0.16251 (8)	0.4165 (2)	0.0456 (5)	
C13	1.03102 (15)	0.19180 (10)	0.3209 (3)	0.0532 (6)	
C14	0.99399 (18)	0.22279 (10)	0.2061 (3)	0.0650 (7)	
H14	1.0318	0.2430	0.1429	0.078*	
C15	0.90212 (19)	0.22521 (11)	0.1802 (3)	0.0671 (7)	
H15	0.8789	0.2469	0.1021	0.081*	
C16	0.84659 (15)	0.19497 (9)	0.2724 (2)	0.0538 (6)	
H16	0.7851	0.1960	0.2554	0.065*	
C17	1.13038 (15)	0.18931 (13)	0.3465 (3)	0.0768 (8)	
H17A	1.1502	0.1519	0.3392	0.115*	
H17B	1.1442	0.2034	0.4464	0.115*	
H17C	1.1603	0.2111	0.2705	0.115*	

### Atomic displacement parameters (Å^2^)

**Table:**

	*U*^11^	*U*^22^	*U*^33^	*U*^12^	*U*^13^	*U*^23^
O1	0.0396 (8)	0.0589 (10)	0.0462 (9)	0.0034 (7)	−0.0010 (6)	0.0099 (7)
O2	0.0318 (8)	0.0918 (13)	0.0569 (10)	−0.0080 (8)	0.0014 (7)	0.0109 (8)
O3	0.0378 (8)	0.0921 (13)	0.0459 (9)	−0.0056 (8)	−0.0042 (7)	0.0152 (8)
O4	0.0370 (9)	0.0835 (12)	0.0597 (10)	0.0003 (8)	−0.0035 (7)	0.0124 (8)
N1	0.0348 (10)	0.0580 (12)	0.0463 (10)	−0.0030 (8)	−0.0004 (8)	0.0027 (8)
N2	0.0375 (10)	0.0623 (12)	0.0422 (10)	−0.0061 (9)	−0.0030 (8)	0.0055 (8)
C1	0.0371 (11)	0.0444 (12)	0.0390 (11)	−0.0017 (9)	−0.0027 (9)	−0.0038 (9)
C2	0.0373 (11)	0.0444 (11)	0.0372 (11)	−0.0011 (9)	−0.0012 (9)	0.0006 (9)
C3	0.0377 (12)	0.0543 (13)	0.0409 (11)	−0.0030 (10)	0.0018 (9)	−0.0034 (10)
C4	0.0520 (14)	0.0595 (15)	0.0448 (12)	−0.0075 (11)	0.0059 (11)	0.0020 (10)
C5	0.0616 (15)	0.0590 (14)	0.0403 (12)	0.0016 (12)	−0.0011 (11)	0.0102 (10)
C6	0.0470 (13)	0.0505 (13)	0.0445 (12)	0.0015 (10)	−0.0070 (10)	0.0015 (10)
C7	0.0754 (19)	0.0632 (18)	0.0787 (17)	0.0105 (14)	−0.0018 (15)	0.0187 (14)
C8	0.0411 (14)	0.102 (2)	0.0677 (16)	−0.0099 (13)	0.0149 (11)	−0.0004 (14)
C9	0.0368 (12)	0.0531 (13)	0.0436 (12)	0.0003 (9)	−0.0030 (9)	0.0007 (10)
C10	0.0348 (11)	0.0501 (12)	0.0437 (12)	0.0008 (10)	−0.0017 (9)	−0.0012 (9)
C11	0.0410 (12)	0.0432 (12)	0.0422 (11)	−0.0039 (9)	−0.0031 (9)	−0.0042 (9)
C12	0.0440 (13)	0.0456 (12)	0.0472 (12)	−0.0001 (10)	−0.0006 (10)	−0.0079 (10)
C13	0.0461 (13)	0.0532 (14)	0.0602 (14)	−0.0050 (11)	0.0092 (11)	−0.0077 (11)
C14	0.0723 (18)	0.0547 (15)	0.0680 (16)	−0.0191 (13)	0.0149 (14)	0.0024 (12)
C15	0.0768 (19)	0.0569 (16)	0.0678 (16)	−0.0098 (13)	−0.0064 (14)	0.0206 (12)
C16	0.0519 (13)	0.0512 (14)	0.0584 (14)	−0.0029 (11)	−0.0090 (11)	0.0061 (11)
C17	0.0450 (15)	0.087 (2)	0.098 (2)	−0.0091 (14)	0.0176 (14)	−0.0022 (16)

### Geometric parameters (Å, °)

**Table:**

O1—C2	1.372 (2)	C7—H7A	0.9600
O1—C7	1.414 (3)	C7—H7B	0.9600
O2—C3	1.370 (2)	C7—H7C	0.9600
O2—C8	1.425 (3)	C8—H8A	0.9600
O3—C10	1.240 (2)	C8—H8B	0.9600
O4—C12	1.349 (2)	C8—H8C	0.9600
O4—H4	0.8195	C9—H9	0.9300
N1—C9	1.273 (3)	C10—C11	1.482 (3)
N1—N2	1.384 (2)	C11—C16	1.394 (3)
N2—C10	1.343 (2)	C11—C12	1.405 (3)
N2—H2A	0.9005	C12—C13	1.398 (3)
C1—C6	1.391 (3)	C13—C14	1.372 (3)
C1—C2	1.398 (3)	C13—C17	1.501 (3)
C1—C9	1.453 (3)	C14—C15	1.391 (4)
C2—C3	1.401 (3)	C14—H14	0.9300
C3—C4	1.378 (3)	C15—C16	1.370 (3)
C4—C5	1.383 (3)	C15—H15	0.9300
C4—H4A	0.9300	C16—H16	0.9300
C5—C6	1.368 (3)	C17—H17A	0.9600
C5—H5	0.9300	C17—H17B	0.9600
C6—H6	0.9300	C17—H17C	0.9600
			
C2—O1—C7	115.99 (16)	O2—C8—H8C	109.5
C3—O2—C8	117.34 (18)	H8A—C8—H8C	109.5
C12—O4—H4	109.3	H8B—C8—H8C	109.5
C9—N1—N2	113.41 (17)	N1—C9—C1	121.57 (19)
C10—N2—N1	119.45 (17)	N1—C9—H9	119.2
C10—N2—H2A	119.4	C1—C9—H9	119.2
N1—N2—H2A	121.1	O3—C10—N2	122.05 (19)
C6—C1—C2	119.12 (18)	O3—C10—C11	121.49 (18)
C6—C1—C9	122.34 (18)	N2—C10—C11	116.46 (17)
C2—C1—C9	118.53 (18)	C16—C11—C12	118.3 (2)
O1—C2—C1	119.28 (17)	C16—C11—C10	122.42 (19)
O1—C2—C3	120.71 (17)	C12—C11—C10	119.17 (18)
C1—C2—C3	119.90 (18)	O4—C12—C13	116.7 (2)
O2—C3—C4	125.12 (18)	O4—C12—C11	122.34 (19)
O2—C3—C2	114.99 (18)	C13—C12—C11	120.9 (2)
C4—C3—C2	119.87 (19)	C14—C13—C12	117.9 (2)
C3—C4—C5	119.7 (2)	C14—C13—C17	122.0 (2)
C3—C4—H4A	120.2	C12—C13—C17	120.1 (2)
C5—C4—H4A	120.2	C13—C14—C15	122.6 (2)
C6—C5—C4	121.1 (2)	C13—C14—H14	118.7
C6—C5—H5	119.4	C15—C14—H14	118.7
C4—C5—H5	119.4	C16—C15—C14	118.6 (2)
C5—C6—C1	120.25 (19)	C16—C15—H15	120.7
C5—C6—H6	119.9	C14—C15—H15	120.7
C1—C6—H6	119.9	C15—C16—C11	121.5 (2)
O1—C7—H7A	109.5	C15—C16—H16	119.2
O1—C7—H7B	109.5	C11—C16—H16	119.2
H7A—C7—H7B	109.5	C13—C17—H17A	109.5
O1—C7—H7C	109.5	C13—C17—H17B	109.5
H7A—C7—H7C	109.5	H17A—C17—H17B	109.5
H7B—C7—H7C	109.5	C13—C17—H17C	109.5
O2—C8—H8A	109.5	H17A—C17—H17C	109.5
O2—C8—H8B	109.5	H17B—C17—H17C	109.5
H8A—C8—H8B	109.5		

### Hydrogen-bond geometry (Å, °)

**Table:**

*D*—H···*A*	*D*—H	H···*A*	*D*···*A*	*D*—H···*A*
O4—H4···O3	0.82	1.91	2.630 (2)	146
N2—H2A···O3^i^	0.90	2.17	3.030 (2)	158

Symmetry codes: (i) −*x*+3/2, *y*, *z*−1/2.
